# Supplementary material for: Expanding the Structural Diversity of DNA Methyltransferase Inhibitors
Source: Pharmaceuticals (Basel). 2020 Dec 27;14(1):17. doi: 10.3390/ph14010017 (PMC7824300; doi:10.3390/ph14010017)
Supplement: Supplementary file 1 [file pharmaceuticals-14-00017-s001.pdf]

SUPPLEMENTARY MATERIAL

Expanding the Structural Diversity of DNA Methyltransferase Inhibitors

K. Eurídice Juárez-Mercado[1], Fernando D. Prieto-Martínez[1], Norberto Sánchez-Cruz[1], Andrea Peña-Castillo[1], Diego Prada-Gracia[2], José L. Medina-Franco*[,1]

[1] DIFACQUIM research group, Department of Pharmacy, School of Chemistry, National Autonomous University of Mexico, Avenida Universidad 3000, Mexico City 04510, Mexico; kaeuridice@gmail.com (K.E.J-M.); ferdpm4@hotmail.com (F.D.P-M.); norberto.sc90@gmail.com (N.S-C,); andrea.pecas93@gmail.com (A.P-C.).

[2] Research Unit on Computational Biology and Drug Design, Children's Hospital of Mexico Federico Gomez, Mexico City, Mexico; prada.gracia@gmail.com (D.P-G.)

***** Correspondence: medinajl@unam.mx

Contents

		Page
**Figure S1**	Chemical structures of the ten most frequent (Bemis-Murcko) scaffolds of the active DNMT inhibitors available in ChEMBL 27.	S2
**Table S1**	Chemical vendors of the ten compounds tested and their purity as supplied by the vendor.	S3
**Figure S2**	Comparison of the co-crystal position of SAH in the catalytic site of DNMT1 (PDB ID: 4WXX) with the predicted binding mode of **A**) **CSC027694519** and **B**) **7936171**.	S4
**Figure S3**.	Docking poses of glyburide with DNMT1 obtained with the software PLANTS.	S5
**Figure S4**	Microstate network analysis of glyburide.	S5

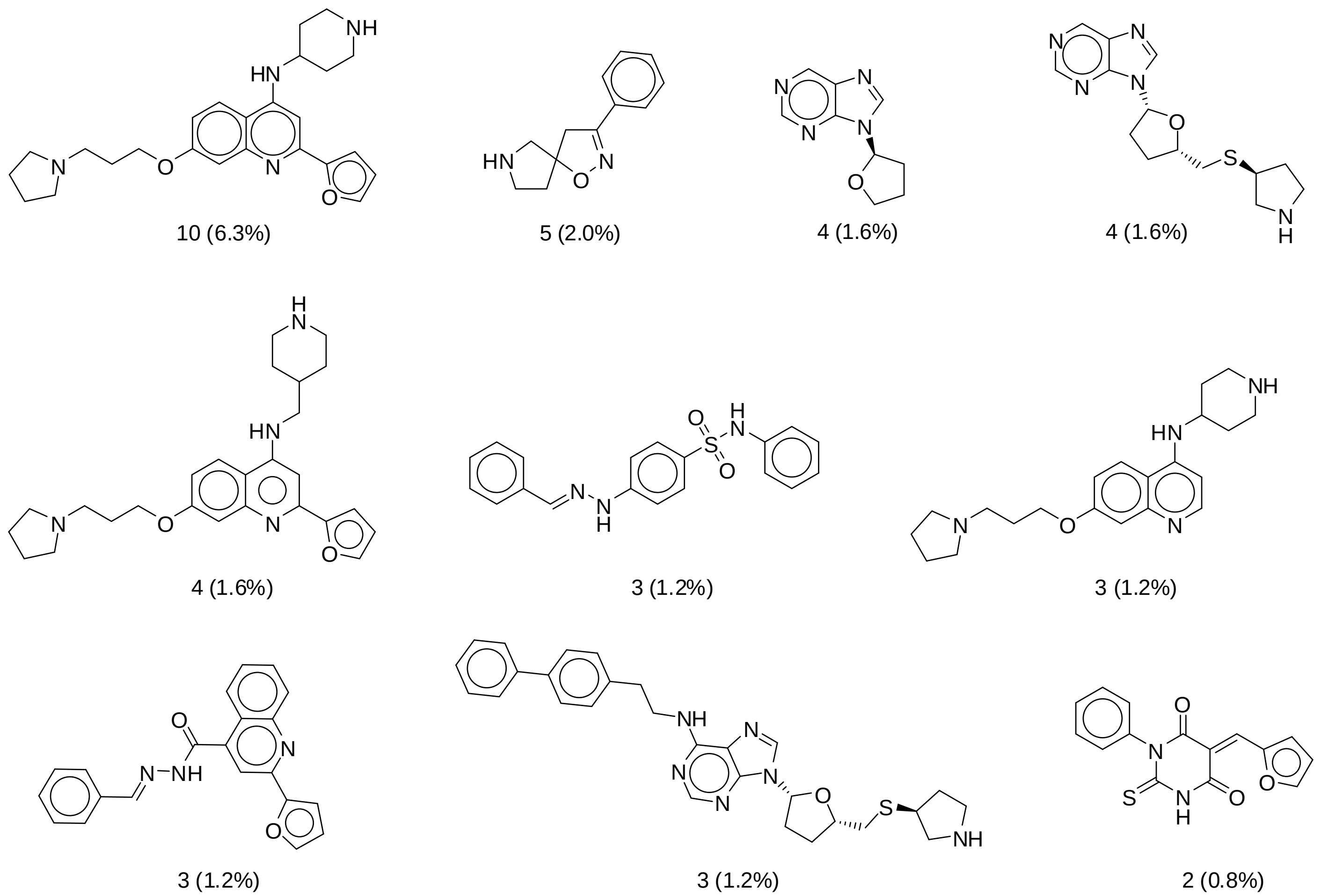

Figure S1. Chemical structures of the ten most frequent (Bemis-Murcko) scaffolds of the active DNMT inhibitors available in ChEMBL 27. The percentage of frequency is indicated in parenthesis.

Table S1. Chemical vendors of the ten compounds tested and their purity as supplied by the vendor.

Compound	Vendor	Purity % (provided by vendor)
Glyburide	TargetMol	99.77
Panobinostat	TargetMol	98
Theaflavin	TargetMol	97.76
**7936171**	Chembridge	>=90
**CSC027480404**	ChemSpace	90
**CSC026286840**	ChemSpace	100
**CSC027694519**	ChemSpace	100
**6631802**	Chembridge	>=90
**CSC027796832**	ChemSpace	94
**CSC027083851**	ChemSpace	100

A

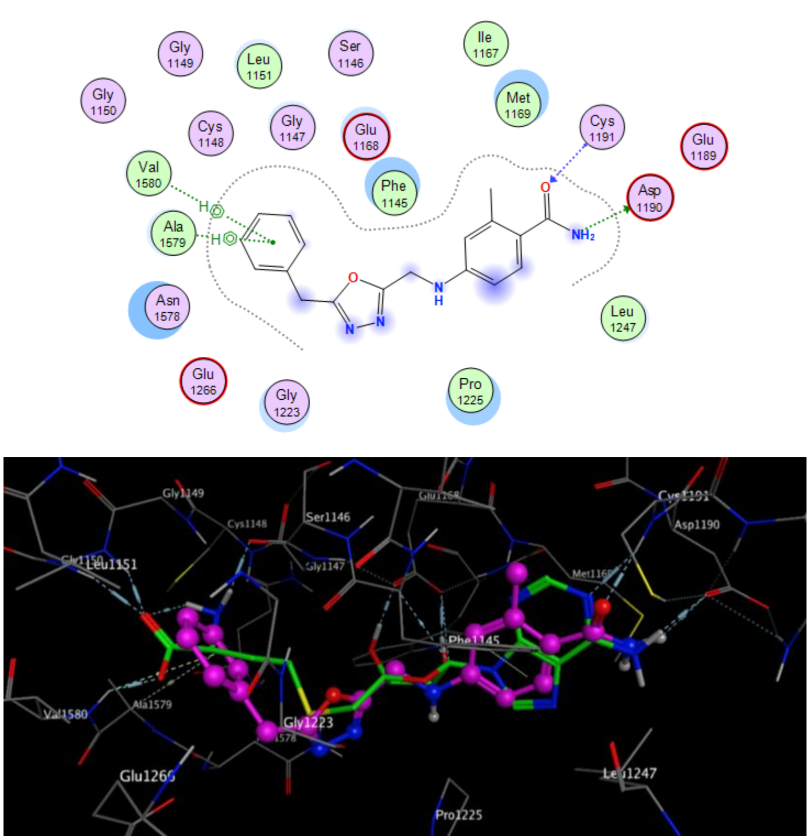

B

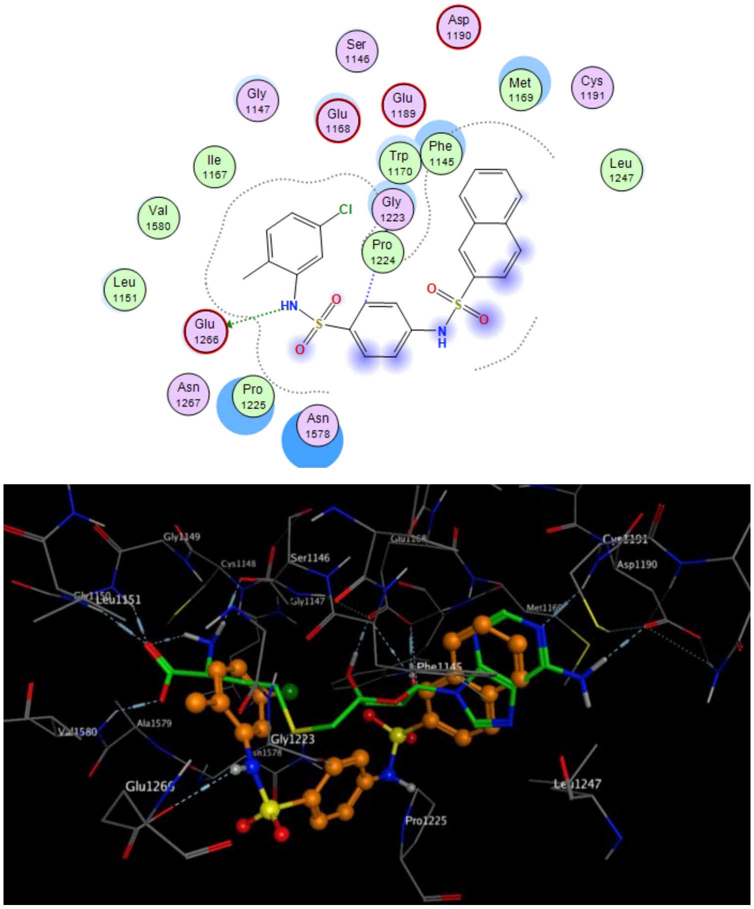

Figure S2. Comparison of the co-crystal position of SAH in the catalytic site of DNMT1 (PDB ID: 4WXX) with the predicted binding mode of **A**) **CSC027694519** and **B**) **7936171**.

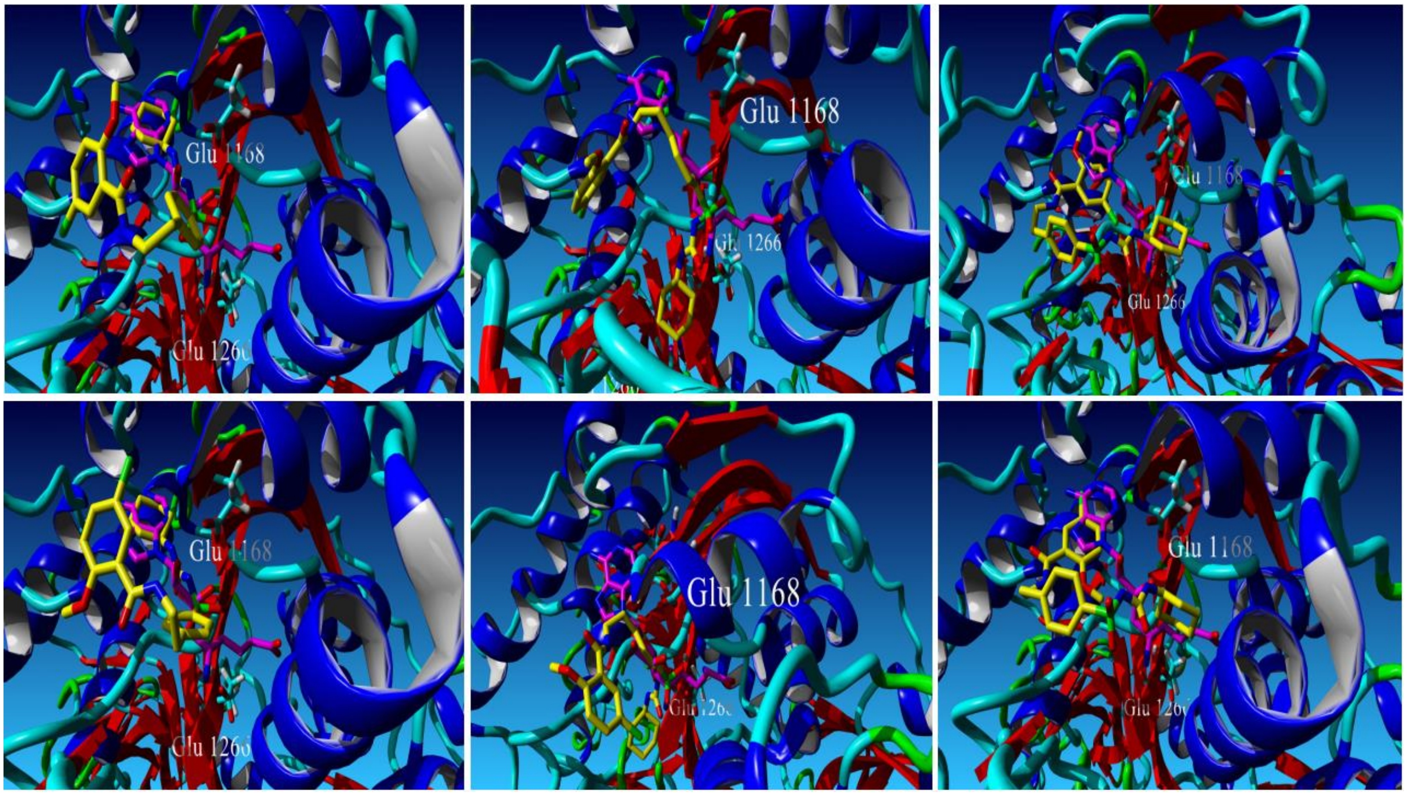

Figure S3. Docking poses of glyburide with DNMT1 obtained with the software PLANTS.

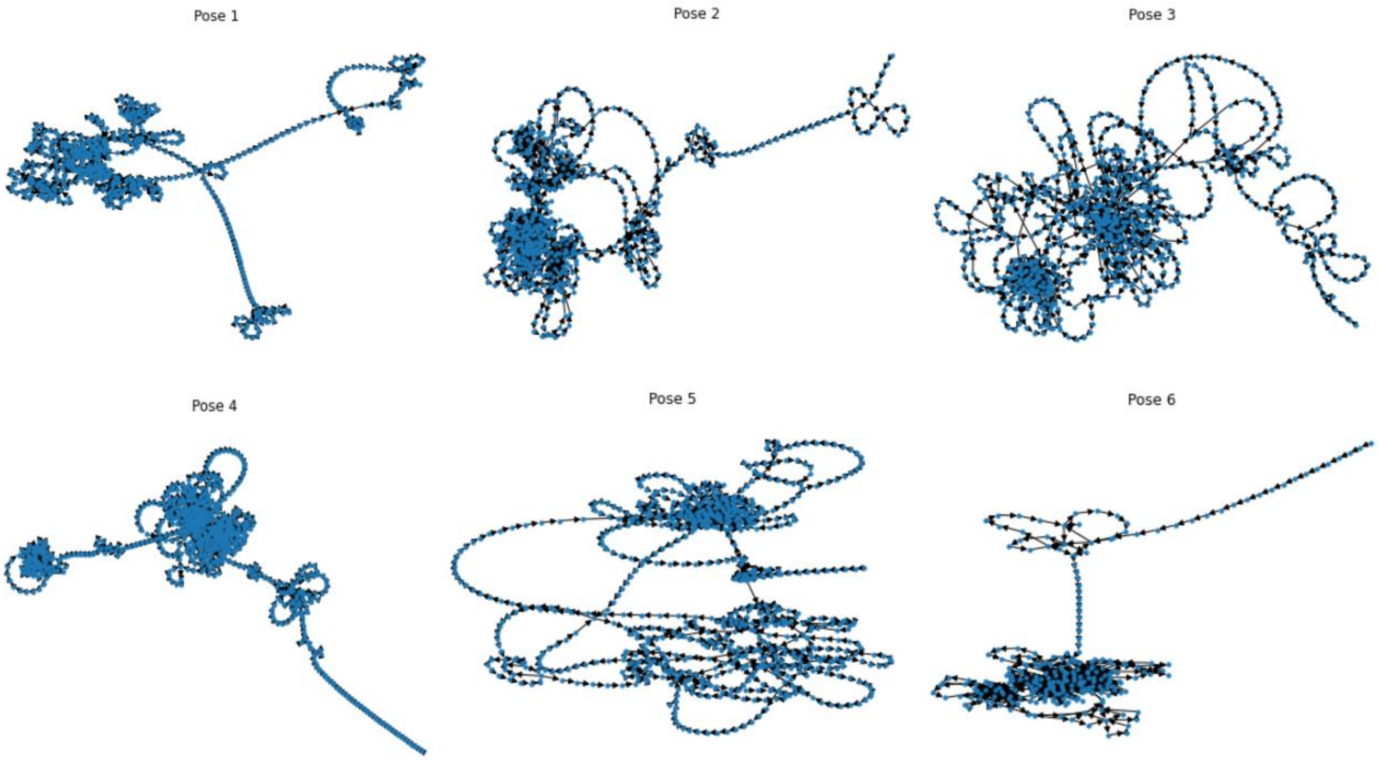

Figure S4. Microstate network analysis of glyburide.
